# A heterotrimeric G protein (Gsα) biomarker may predict antidepressant response in subjects with major depressive disorder

**DOI:** 10.3389/fpsyt.2025.1619243

**Published:** 2025-07-18

**Authors:** Steven D. Targum, Aksu Gunay, Alex Leow, Olusola A. Ajilore, Mark H. Rapaport, Mark M. Rasenick

**Affiliations:** ^1^ Department of Psychiatry, University of Illinois College of Medicine, Chicago, IL, United States; ^2^ Department of Psychiatry, Pax Neuroscience, Glenview, IL, United States; ^3^ Department of Psychiatry, University of Utah School of Medicine, Chicago, IL, United States; ^4^ Department of Psychiatry, Jesse Brown Veteran's Administration Medical Center (VAMC), Chicago, IL, United States

**Keywords:** antidepressants, major depressive disorder, biomarkers, heterotrimeric G protein, GPCR: lipid raft: prediction of treatment response

## Abstract

**Background:**

The disproportionate sequestration of the heterotrimeric G protein (Gsα) in lipid raft regions during acute depressive episodes can impair neurotransmitter signaling by restricting its interaction with and activation of adenylate cyclase and consequently reduce cyclic adenosine monophosphate (cAMP) production. In humans, Gsα is measured as a peripheral biomarker from platelet samples by using prostaglandin-1 (PGE-1) to stimulate adenylyl cyclase. In two previous studies, Gsα biomarker responses were significantly lower in acutely depressed subjects with major depressive disorder (MDD) than healthy controls and were correlated with the magnitude of symptom severity.

**Methods:**

The potential utility of Gsα biomarker responses to anticipate antidepressant treatment (ADT) response was assessed in 19 acutely depressed MDD subjects receiving ADT for 6 weeks.

**Results:**

Following 6 weeks of ADT, Gsα biomarker responses increased significantly in 11 ADT responders compared with 8 non-responders (Mann–Whitney U test; p= 0.033), particularly in subjects with the lowest Gsα biomarker values at screen. All five MDD subjects with Gsα biomarker screen values<1.5 nM cAMP/well became ADT responders with mean Gsα biomarker responses increasing >100% at 6 weeks in contrast to 10% in subjects with higher screen values (p= 0.012).

**Conclusion:**

ADT facilitates translocation of Gsα from the lipid raft region, particularly in MDD subjects who respond to ADT. The findings from this small hypothesis-generating study suggest that the Gsα biomarker assay has potential clinical utility to predict ADT response in depressed subjects with low baseline biomarker values. However, these are exploratory findings that must be replicated in larger studies.

## 1 Introduction

Major depressive disorder (MDD) is a heterogeneous syndrome characterized by a variety of clinical presentations and symptoms that generate substantial medical, economic, and social costs (1–6). Unfortunately, antidepressant treatment (ADT) is not always effective and may require several weeks to work, and nearly one-third of adequately treated subjects do not achieve remission (7). Given the considerable burden caused by MDD, there is a clear need for a practical and quantitative method to differentiate and optimize treatment options as early as possible. Currently, there is no clinical tool that can determine which ADT will be most effective for a specific individual (8–10). A simple and easily obtained biomarker that might facilitate medication decisions would be a useful tool in treatment planning for individuals with MDD.

The heterotrimeric G protein (Gsα) has been explored as a therapeutic target for several disease entities including depression (11–13). We have explored the utility of Gsα as a simple protein biomarker in individuals with acute MDD where it appears to be sensitive to symptomatic change following ADT (14, 15). Gsα is normally distributed between two membrane regions: non-raft regions and a specialized region called the lipid raft that is associated with cytoskeletal elements and is rich in cholesterol (16, 17). It has been shown that the distribution of Gsα is skewed during acute depressive episodes and becomes more concentrated in the lipid raft region, apparently anchored by the structural protein tubulin (18–21). This disproportionate sequestration of Gsα in lipid raft regions impairs neurotransmitter signaling by restricting its interaction with and activation of AC and consequently reduces cAMP production (21). Preclinical studies have shown that several approved antidepressants with different mechanisms of action can increase Gsα signaling and evoke translocation of Gsα from lipid rafts (22–25). The subsequent, enhanced interaction of Gsα with the effector enzyme adenylyl cyclase (AC) stimulates its enzymatic activity and leads to an increase in the production of cyclic adenosine monophosphate (cAMP). There is substantial evidence that cAMP signaling is involved in antidepressant action and that the long-term sequelae of ADT may be associated with sustained cAMP transmission as well as cAMP-induced transcription of growth factor genes (26–29).

**Figure 1:**
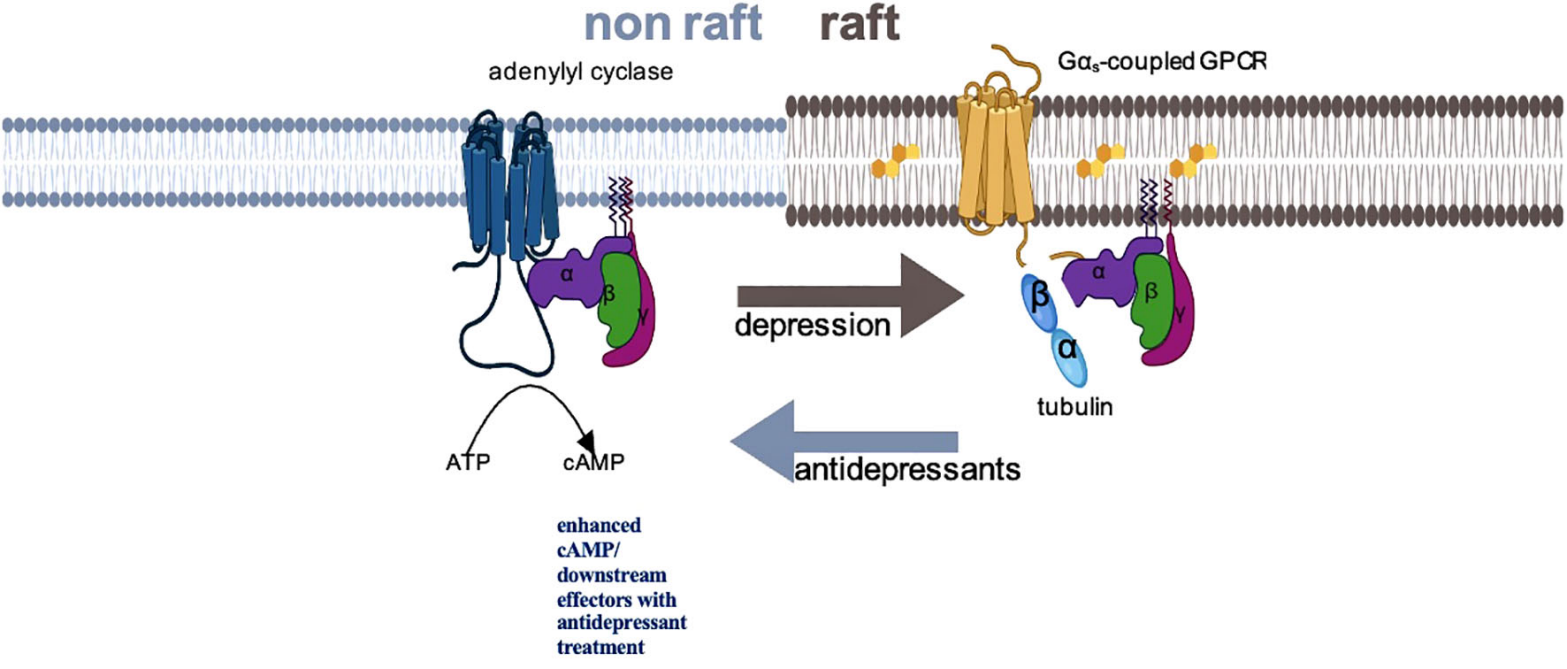
Schematic representation of Gsα disposition in depression and with antidepressant treatment. In depressed subjects, Gsα is disproportionately localized in lipid raft fractions of the membrane, where the more rigid structure dampens mobility of that protein, preventing interaction with adenylyl cyclase. Successful antidepressant treatment displaces Gsα from lipid rafts, facilitating interaction with adenylyl cyclase and augmenting cAMP signaling.

The identification of this specific molecular pathway in preclinical studies has facilitated the exploration of a potential Gsα biomarker in individuals with MDD. **Figure 1** provides a proposed schematic representation of the disposition of Gsα during acute depressive episodes and following ADT. In humans, Gsα can be measured as a peripheral proxy from white blood cells or platelet samples by using prostaglandin-1 (PGE-1), an agonist for Gsα-coupled GPCRs to stimulate adenylyl cyclase (25, 30–33).

In two small clinical studies, we examined the relationship of the Gsα biomarker to symptom severity in MDD subjects and healthy controls (14, 15). In both studies, Gsα biomarker responses distinguished acutely depressed subjects from healthy controls and were correlated with the magnitude of symptom severity within the MDD group. The first study assessed changes in Gsα biomarker responses in MDD subjects following 6 weeks of ADT (14). The second study assessed the reliability of the Gsα biomarker in MDD subjects and explored the utility of Gsα biomarker response thresholds to differentiate between MDD subjects and healthy controls (15). In this report, we revisited the first study to explore whether the Gsα biomarker thresholds identified in the second study could serve as predictors of ADT response.

## 2 Materials and methods

Subjects and data for this report come from a 6-week open-label ADT study conducted at the Emory University School of Medicine Mood and Anxiety Disorders Program between September 2013 and May 2016 (14). The study was reviewed and approved by the institutional review board of the Emory University School of Medicine. All study participants signed an IRB-approved consent to participate and consent to give blood samples. All participating subjects were compensated for their participation in the study. The study was conducted in accordance with the Declaration of Helsinki (1964) and Good Clinical Practices as outlined by the International Conference on Harmonization (1997).

Full eligibility criteria are presented elsewhere (14). The study recruited depressed subjects with non-psychotic MDD and healthy controls. Eligible MDD subjects met DSM-IV TR criteria for MDD based upon the Structured Clinical Interview for DSM-IV Axis I Disorders-Patient Edition (SCID-I/P) and had a score ≥15 on the Hamilton rating scale for depression (HamD_17_) at the screen visit (34–36). The DSM-IV TR criterion was the diagnostic criteria used in the United States at the time this study was approved by the institutional review board in 2013. Eligible depressed subjects had not been taking antidepressant or other psychotropic medications (except for sedatives) for at least 4 weeks prior to the initiation of ADT. Healthy controls had no history of depression and had HamD_17_ scores ≤1. Clinic visits included a screen and 6-week visit that followed open-label ADT for participating MDD subjects. Whole blood for Gsα marker analysis was collected at screen and baseline in all participants and 6 weeks for subjects receiving ADT.

### 2.1 Preparation and analysis of Gsα biomarker samples

The blood samples were collected without regard to fasting or time of day. After each blood draw, blood samples were centrifuged in a 10-mL EDTA collection tube at 500 × *g* for 5 min at 4°C. The platelet-rich plasma layer was transferred into 15-mL conical tubes. Subsequently, the platelet samples were centrifuged in 15-mL conical tubes at 2,000 x *g* for 5 min at 4°C. Platelet pellets were resuspended in TEM buffer (10 mM Tris HCl, 1 mM Mg Cl_2_, EDTA pH 7.5, protease inhibitor cocktail, Sigma # P2714), frozen, and stored at −80°C. Prior to assay, samples were thawed. A BCA protein assay was conducted, and the concentration of platelet suspensions was adjusted to 1 µg/mL for the adenylyl cyclase assay as triplicates. The PerkinElmer AlphaScreen cAMP assay kit was performed with a 384-well plate following the manufacturer’s directions. The acceptor beads were added in the stimulation buffer (1mM HEPES pH 7.5, 500µM IBMX, 0.1% BSA, 25 mM MgCl_2_, 375 mM NaCl, 250 mM ATP, 2.5 mM GDP, and 2.5 nM GTP in HBSS). Subsequently, a 5-µL total volume of cells/beads was added to each well as triplicates. Adenylyl cyclase activity was measured both without a stimulating agent (basal, 5 µL stimulation buffer) and in the presence of 10 µM prostaglandin E1 (PGE1) in 5 µL of stimulation buffer. The 384-well plate was incubated for 30 min at RT to allow cAMP accumulation. The reaction was stopped by adding 15 µL of 1.67xbiotin-cAMP/Streptavidin Donor Bead Detection Mix. The plate was sealed and kept in the dark overnight. Plates were read on a Molecular Devices SpectraMax i3x plate reader. The cAMP produced was calculated from a standard curve run with each assay. The PGE1 stimulation cAMP response as reported in this paper reflects the ratio of PGE1 stimulation of adenylyl cyclase (AC) activity normalized over basal AC activity (expressed as cAMP response). More details about the preparation and analysis of Gsα biomarker samples are provided in previous publications (10, 11).

The Gsα biomarker response as reported in this paper reflects the ratio of PGE-1 stimulation of adenylyl cyclase (AC) activity normalized over basal AC activity (expressed as nM cAMP/well). This measure has been proven to be quite reliable with consistent values that varied by no more than 5% within 2 weeks (11).

### 2.2 Data analyses

The relationship of Gsα biomarker responses to ADT response following 6 weeks of treatment was assessed using HamD_17_. The utility of Gsα biomarker response cutoff screen thresholds of<1.5 and<1.8 were used to examine the prediction of ADT response based upon the *post-hoc* analyses derived from the UnMASCK study (Unobtrusive Monitoring of Affective Symptoms and Cognition Using Keyboard Dynamics study of mood disorders: NCT04358900) conducted at the University of Illinois Chicago (15).

Subjects were stratified into ADT treatment responders and non-responders based upon a criterion of ≥50% HamD_17_ score improvement from the screen visit (37). A value of >30% change of the Gsα biomarker response between screen and week 6 was used as the criterion for Gsα biomarker response based on our previous report (10). This was a small exploratory study, and power analyses were not done regarding sample size. Statistical analyses included Student’s t test, Fisher exact test, and the Mann–Whitney U test as a non-parametric statistical tool for the analysis of Gsα biomarker thresholds with different distributions.

## 3 Results

Data were available for 19 treated MDD subjects at both the screen and week 6 visits. The antidepressants prescribed were escitalopram (7), citalopram (4), fluoxetine (3), duloxetine (2), venlafaxine XR (2), and nortriptyline (1).

There were no significant demographic or ADT differences between the treatment responders and non-responders. As shown in **Table 1**, there were 11 ADT responders and 8 non-responders.

**Table 1:** Gsα biomarker responses after 6 weeks of antidepressant treatment in the Emory study*.

	n	HamD_17_ screen	HamD_17–_6 weeks	Responders	Gsα biomarker at screen	Gsα biomarker percent change from screen**
All subjects	19	20.3	9.3		2.95	34.0%
ADT responders	11	20.4	5.7	11 (58%)	2.20	62.0%
ADT non-responders	8	20.1	14.1	8 (42%)	3.99	-4.6%
T test/Mann–Whitney U		t= 0.19	t= -4.56		t= -1.44	z= 2.14
p (responders vs. non-responders)		p= 0.85	p= 0.0003		p= 0.168	p= 0.033

*Gsα biomarker response indicates the change of prostaglandin (PGE-1)-stimulated adenylyl cyclase activity (normalized over basal activity) expressed as nM cAMP/well. Treatment response is defined as ≥50% improvement of the screen HamD_17_ score after 6 weeks of antidepressant treatment.

** Gsα marker percent change from screen reflects the ratio of biomarker change relative to Gsα marker response at screen

The mean screen Gsα biomarker responses were 2.20 ± 1.2 nM cAMP/well at the screen visit in the 11 ADT responders compared with 3.99 ± 3.9 nM cAMP/well in the 8 non-responders (F= 2.06; p=0.169). After 6 weeks of treatment, the mean Gsα biomarker response was 3.55 ± 3.1 nM cAMP/well in the ADT responders (a 62.0% mean increase from the screen assessment) and 3.67 ± 4.2 in the non-responder cohort, reflecting a mean 4.6% decrement from the screen value (Mann–Whitney U test; n_a_= 11, n_b_= 8; z= 2.14; p= 0.033; effect size= 0.48). Thus, the mean Gsα biomarker value of the ADT responders was low at screen but increased significantly and was essentially equivalent to the non-responder values after 6 weeks. Individually, 8 of the 11 ADT responders (72.7%) had a >30% increase of the screen Gsα biomarker response in contrast to two of eight non-responders (25%) following 6 weeks of ADT (Fisher exact test= 0.07).

As shown in **Table 2**, 8 of the 19 MDD subjects had Gsα biomarker responses<1.8 nM cAMP/well at the screen visit. After 6 weeks of ADT, the mean percentage increase of Gsα biomarker responses was significantly greater in the MDD subjects with Gsα marker values<1.8 at screen compared with subjects with values >1.8 (Mann–Whitney U test; n_a_= 11, n_b_= 8; z= −2.91; p= 0.004; effect size= 0.63). Using a threshold of<1.8 at the screen visit, the Gsα biomarker response increased >30% in 8 of the 8 subjects (100%) by week 6 in contrast to 2 of the remaining 11 subjects (18.1%) with screen values >1.8 (Fisher exact test= 0.003).

**Table 2:** Screen thresholds and Gsα biomarker responses after 6 weeks of antidepressants*.

	n	HamD_17_ screen	HamD_17–_6 weeks	Responders	Gsα biomarker at screen	Gsα biomarker percent change from screen**
Gsα marker response<1.8 at screen	8	20.5	9.0	6 (75%)	1.45	81.5%
Gsα marker response >1.8 at screen	11	20.1	9.5	5 (45%)	4.04	-0.6%
Statistical analysis		t= 0.33	t= -0.17		t= -2.24	z= -2.91
p Gsα marker response<1.8 or >1.8		p= 0.75	p= 0.87	p= 0.35***	p= 0.039	p=0.004
Gsα marker response<1.5 at screen	5	19.4	4.6	5 (100%)	1.36	100.8%
Gsα marker response >1.5 at screen	14	20.6	10.9	6 (43%)	3.52	10.1%
Statistical analysis		t= -0.85	t= -2.37		t= -1.56	z= -2.50
p Gsα marker response<1.5 or >1.5		p= 0.41	p= 0.030	p= 0.045***	p= 0.137	p= 0.012

*Gsα biomarker response indicates the change of prostaglandin (PGE-1)-stimulated adenylyl cyclase activity (normalized over basal activity) expressed as nM cAMP/well. Treatment response is defined as ≥50% improvement of the screen HamD_17_ score after 6 weeks of antidepressant treatment.

** Gsα marker percent change from screen reflects the ratio of biomarker change relative to Gsα marker response at screen. Calculation used was non-parametric Mann–Whitney U test.

***Fisher exact test

Five MDD subjects had Gsα biomarker responses<1.5 nM cAMP/well at the screen visit. These five subjects received SSRIs that included citalopram (2), escitalopram (2), and fluoxetine (1).

After 6 weeks of ADT, the mean Gsα biomarker response increased from 1.36 ± 0.16 to 2.77 ± 0.98 (>100% increase) in these five subjects in contrast to 3.52 ± 0.14 to 3.89 0.14 (10% increase) in the MDD subjects with screen Gsα biomarker responses >1.5 (Mann–Whitney U test; n_a_= 14, n_b_= 5; z= −2.50 p= 0.012; effect size= 0.55). The Gsα biomarker responses increased >30% by week 6 in all five ADT responders (100%) who had screen values<1.5 in contrast to 5 of the 14 subjects (36.6%) whose Gsα biomarker responses were >1.5 at the screen visit (Fisher exact test= 0.03). In this small sample, all five MDD subjects with screen Gsα biomarker responses<1.5 nM cAMP/well became ADT responders in contrast to 6 of the 14 other subjects with higher screen values (Fisher exact test= 0.045).

## 4 Discussion

Many studies have sought useful biomarkers to facilitate the diagnosis and/or treatment of MDD (8–10, 38). We have explored the extent of lipid-raft localization of the heterotrimeric G protein (Gsα) as a potential biomarker in MDD. In two clinical studies, we found that this peripheral Gsα biomarker was significantly lower in acutely depressed MDD subjects than healthy controls and inversely correlated with symptom severity (14, 15). In this paper, we report a new analysis of the initial (Emory) study data, which focused on the potential utility of the Gsα biomarker to predict ADT response using biomarker response thresholds identified in the UnMASCK study (15).

The mean Gsα biomarker response increased significantly from the screen value in the 11 ADT responders versus 8 non-responders (p=0.033). The mean Gsα biomarker value of the ADT responders was low at screen but increased significantly and was essentially equivalent to the non-responder values at 6 weeks.

Both Gsα biomarker response thresholds assayed at the screen visit differentiated the ADT responders from non-responders. After 6 weeks of ADT, the mean percentage increase of Gsα biomarker responses was significantly greater in the MDD subjects with screen threshold values of either<1.5 and<1.8 nM cAMP/well compared with subjects with higher screen values (p= 0.012 and 0.004, respectively). The individual Gsα biomarker responses increased >30% in all of the low threshold (<1.5 nM cAMP/well) subjects (100%) in contrast to 36.7% and 18.1% of the subjects with screen values >1.5 and >1.8, respectively (Fisher exact test: p= 0.03 and 0.003, respectively). Furthermore, all five MDD subjects with Gsα biomarker values<1.5 at screen became ADT responders and yielded mean Gsα biomarker responses that increased >100% in contrast to 10% in subjects with higher screen biomarker values (p= 0.011). This latter finding suggests that a low pretreatment Gsα biomarker value that increases after the initiation of ADT may anticipate treatment response in some depressed patients. Clearly, larger studies examining the Gsα marker response shortly after the initiation of ADT are needed to explore this possibility.

The clinical findings of a robust increase of Gsα biomarker responses following the initiation of ADT is consistent with preclinical findings that Gsα translocation from the lipid raft region is facilitated by various classes of antidepressants (38–46). In preclinical studies, selective serotonin reuptake inhibitors (SSRIs), serotonin–norepinephrine reuptake inhibitors, tricyclic antidepressants, monoamine oxidase inhibitors, and ketamine all increased Gsα signaling and evoke translocation of Gsα from lipid rafts (22–24, 38–46). The findings are also consistent with a PET imaging study using ^11^C-(R)-rolipram that found decreased cAMP levels in brain scans of unmedicated MDD patients increased after 8 weeks of SSRI treatment (46).

Our findings must be interpreted with caution. First, both studies used small sample populations and did not include double-blind placebo-controls for differential analysis of biomarker changes. Second, it must be acknowledged that the choice of Gsα biomarker response thresholds and change criterion (>30%) were chosen in a *post-hoc* fashion and derived from small study samples. The risk of type 1 error is elevated in analyses of small samples like this, and larger studies are needed to replicate and clarify these criteria. Third, the depressed subjects in this open-label study were treated with a variety of antidepressants and it is not known if different antidepressants might yield different Gsα biomarker responses in humans. Numerous preclinical studies have shown that there is little if any variation of Gsα biomarker responses regardless of the antidepressant selected, including ketamine, whereas antipsychotics, anxiolytics, and mood stabilizers do not affect Gsα biomarker responses (22, 23, 44, 45). Nonetheless, more studies are needed to elucidate the effect, if any of different ADT on the Gsα biomarker response in acutely depressed subjects. Fourth, the Gsα biomarker thresholds we explored did not identify all acutely depressed subjects or exclude all healthy controls (11). Clearly, the heterogeneity of depressive disorder is a confounding factor that may affect sensitivity in this population (4–6). It is also possible that individuals with lower Gsα biomarker responses have a greater risk for MDD whether they manifest acute depressive symptoms or not. The apolipoprotein E (APOE) marker is a similar type of risk factor used for dementia of the Alzheimer’s type, and the measurable residual disease (MRD) testing used in oncology reflects the utility of a marker to facilitate treatment planning (47, 48).

MDD is diagnosed primarily by subjective assessments and history without biomarker confirmation, and treatment outcome is often influenced by multiple behavioral and environmental factors that are unrelated to the underlying disease (49–52). Given the complexity of the diagnosis and the heterogeneous nature of the disease, a predictive biomarker of antidepressant response would be extremely useful. It is possible that this Gsα biomarker may be a useful predictor of treatment response for some acutely depressed individuals, particularly subjects who present with low pretreatment Gsα biomarker responses. In humans, the population of circulating platelets turns over approximately every 7 to 8 days (53). Consequently, the Gsα biomarker assay can be repeated after 1 week of ADT to obtain new Gsα response data. Although we have yet to test this hypothesis, it is possible that early changes of the pretreatment Gsα biomarker response might predict eventual treatment success or failure. Gsα biomarker response findings could support treatment decisions regarding continuation of the current antidepressant regimen. Alternatively, different antidepressants might be tested in an ex vivo platform to determine which can increase low pretreatment Gsα marker responses in the symptomatic individuals. Therefore, as a companion to personalized treatment planning, the Gsα biomarker assay may be able to identify the most promising antidepressants for specific depressed individuals. Clearly, these are exploratory and hypothesis-generating findings that require larger studies to understand the potential utility of this protein biomarker assay as a predictive marker to assist the treatment of MDD.

## Data Availability

The raw data supporting the conclusions of this article will be made available by the authors, without undue reservation.
